# A Fast and Sensitive One-Tube SARS-CoV-2 Detection Platform Based on RTX-PCR and *Pyrococcus furiosus* Argonaute

**DOI:** 10.3390/bios14050245

**Published:** 2024-05-13

**Authors:** Rui Han, Fei Wang, Wanping Chen, Lixin Ma

**Affiliations:** 1 State Key Laboratory of Biocatalysis and Enzyme Engineering, Hubei Key Laboratory of Industrial Biotechnology, School of Life Sciences, Hubei University, Wuhan 430062, China; hanrui@stu.hubu.edu.cn (R.H.); wangfei@hubu.edu.cn (F.W.); 2 School of Pharmacy, Qingdao University, Qingdao 266071, China

**Keywords:** nucleic acid detection, thermostable reverse transcriptase, pfAgo, SARS-CoV-2, one-tube

## Abstract

Since SARS-CoV-2 is a highly transmissible virus, alternative reliable, fast, and cost-effective methods are still needed to prevent virus spread that can be applied in the laboratory and for point-of-care testing. Reverse transcription real-time fluorescence quantitative PCR (RT-qPCR) is currently the gold criteria for detecting RNA viruses, which requires reverse transcriptase to reverse transcribe viral RNA into cDNA, and fluorescence quantitative PCR detection was subsequently performed. The frequently used reverse transcriptase is thermolabile; the detection process is composed of two steps: the reverse transcription reaction at a relatively low temperature, and the qPCR performed at a relatively high temperature, moreover, the RNA to be detected needs to pretreated if they had advanced structure. Here, we develop a fast and sensitive one-tube SARS-CoV-2 detection platform based on Ultra-fast RTX-PCR and *Pyrococcus furiosus* Argonaute-mediated Nucleic acid Detection (PAND) technology (URPAND). URPAND was achieved ultra-fast RTX-PCR process based on a thermostable RTX (exo-) with both reverse transcriptase and DNA polymerase activity. The URPAND can be completed RT-PCR and PAND to detect nucleic acid in one tube within 30 min. This method can specifically detect SARS-CoV-2 with a low detection limit of 100 copies/mL. The diagnostic results of clinical samples with one-tube URPAND displayed 100% consistence with RT-qPCR test. Moreover, URPAND was also applied to identify SARS-CoV-2 D614G mutant due to its single-nucleotide specificity. The URPAND platform is rapid, accurate, tube closed, one-tube, easy-to-operate and free of large instruments, which provides a new strategy to the detection of SARS-CoV-2 and other RNA viruses.

## 1. Introduction

Coronavirus (CoV) is a positive-sense single-stranded RNA virus with an envelope named for the similarity between the spinous process and the crown of the viral envelope [1]. It has brought about a huge crisis in human health and the global economy [2,3]. Therefore, early diagnosis of coronavirus is necessary to facilitate subsequent prevention and control measures [4,5]. In the past three years, with the global outbreak of SARS-CoV-2, the establishment of a rapid, accurate, and multi-locus detection platform for SARS-CoV-2 and its mutant strains will greatly reduce the incidence of the virus and provide a rapid response to the outbreak of the pandemic [6]. Nucleic acid detection is aimed at the RNA or DNA detection of viruses. Currently, the nucleic acid detection methods used for COVID-19 mainly include fluorescence quantitative PCR, isothermal amplification technology [7,8], CRISPR technology, such as SHERLOCK [9], SHERLOCKv2 [10], STOPCOVID (SHERLOCK Testing in One Pot for detecting SARS-CoV-2) [11], viral genome sequencing [12,13], and PfAgo detection technology [14,15]. Currently, the most recommended nucleic acid detection is RT-qPCR, the RT-qPCR kits include reverse transcriptase, DNA polymerase, target-specific primers and TaqMan probes [14]. This method has high sensitivity and specificity, but it requires expensive instruments such as fluorescent quantitative PCR system, and time-consuming (1–2 h), which is not conducive to rapid detection [16].

One of the main concerns of RT-PCR is to perform both reverse transcription and DNA polymerization assay in one step in a single tube reaction to avoid cross-contamination between samples. Another crucial aspect of RNA virus detection is the thermostability and reliability of the reverse transcriptase used for cDNA synthesis, especially in one-step assay [17]. RTX (exo-) is a heterogenous polymerase with reverse transcriptase and DNA polymerase activities; it is derived from KOD DNA polymerase of family B from *Thermococcus kodakarensis*. The error rate of RTX (exo-) is 1.0 × 10^−4^, which is comparable to the commercial reverse transcriptase from Moloney murine leukemia virus (MMLV). [18]. Hoffmeisterová et al. developed an RT-PCR assay based on RTX (exo-) to detect Tobacco mosaic virus (TMV), Potato virus X (PVX), and Potato virus Y (PVY) using both total RNA and crude liquid from infected plants [17]. Compared with two-step RT-PCR, single-step RT-PCR does not require frequent switching of the tube to mix with various reagents, which reduces the risk of DNA contamination. The sensitivity was comparable to that of the currently used two-tube RT-PCR with retroviral reverse transcriptase and thermostable DNA polymerase [17]. The research shows that RTX one-step RT-PCR is more effective than the existing two-tube method [19].

*Pyrococcus furiosus* Argonaute (PfAgo) is a DNA-guided DNA cleavage programmable enzyme with high levels of activity at temperatures between 80 °C and 100 °C [20]. In 2019, He et al. developed a PfAgo-mediated nucleic acid detection method (PAND) [21], and Wang et al. simplified PAND and applied it to the detection of SARS-CoV-2 and its variants (SARS-CoV-2 PAND) in 2020 [14], in 2021, Wang et al. proposed a PLCR (PfAgo coupled with modified ligase chain reaction for nucleic acid detection) method to detect SARS-CoV-2 and HPV [15]. To develop a rapid, one-step point-of-care assay, Xun et al. report a scalable and portable assay (SPOT) system that combines reverse transcription-loop-mediated isothermal amplification (LAMP) and PfAgo for field surveillance of multiple SARS-CoV-2 loci [22], which can detect SARS-CoV-2 and its mutants within 30 min. 

The PfAgo digestion system will interfere with the RT-PCR system. The detection method based on RT-PCR and PfAgo has been used to detect SARS-CoV-2, but RT-PCR and detection are not in one tube [14]. It is divided into two steps: the RT-PCR amplification is performed first, then the PfAgo digestion system is performed for detection, which is not only cumbersome to operate but also might easily produce aerosol contamination in the lab. Here, we develop a fast and sensitive one-tube detection platform based on Ultra-fast RTX-PCR and *Pyrococcus furiosus* Argonaute-mediated Nucleic acid Detection (PAND) technology (URPAND) to detect SARS-CoV-2. RTX (exo-) with both thermostable reverse transcriptase and DNA polymerase activity was used to achieve a single-enzyme, one-tube RT-PCR in URPAND, eliminating the need for additional reverse transcription step and shortening the detection time. In addition, the paraffin mixture was used to form a physical barrier to separate the PfAgo cleave mixture from the RT-PCR amplification system to achieve one-tube detection, which could not only simplify the operation process but also reduce the risk of nucleic acid aerosol contamination. The thermostable RTX (exo-) was used in one-tube RT-PCR in URPAND and completed RT-PCR within 25 min; the paraffin mixture melted gradually during the RT-PCR reaction due to the increased temperature, and then the mixture (PfAgo, input guide DNA and fluorescent molecular beacon) fallen into the bottom of the PCR tube by centrifugation, and mixed with the RT-PCR products, fluorescence signal can be observed after reaction for 2 min at 95 °C with Blue Light Transilluminators.

In this study, the URPAND platform was used to detect SARS-CoV-2 in one tube. This platform might shorten detection time, reduce equipment costs, and have a low risk of aerosol contamination. URPAND can detect RNA viruses within 30 min. The detection time, sensitivity, and accuracy of URPAND have advantages compared to the current gold standard RT-qPCR for RNA virus detection. Moreover, one shortcoming of the RT-qPCR test is the high false-negative rate due to the aerosol contamination caused by amplification and accumulation of the target sequence [23]. URPAND is more sensitive and reproducible because amplification of the targets is performed in an RT-PCR system without the interference of TaqMan probes. Therefore, this study developed a nucleic acid detection technology with high sensitivity, specificity, accuracy, low cost, and simple operation, which will provide a new and reliable strategy for nucleic acid detection.

## 2. Materials and Methods

### 2.1. Strains, Plasmids, Chemical Reagents and Samples

*Escherichia coli* BL21(DE3) and DH5α were stored in the laboratory. The pET-28a (+) used as the expression vector was maintained in our laboratory. The plasmid pET-28a (+)-RTX (exo-) coding *Thermococcus kodakarensis* KOD (RTX (exo-) [18] was synthesized by Wuhan Gene Create Biological Engineering Co., Ltd. (Wuhan, China). The plasmid pET-28a (+)-PfAgo coding *Pyrococcus furiosus* Argonaute (PfAgo) was stored in our laboratory. The Eva EZ™ Fluorometric Polymerase Activity Assay Kit was purchased from Biotium (San Francisco, CA, USA). The HiFiScript cDNA Synthesis Kit was purchased from Kangwei Century Biotechnology Co., Ltd. (Taizhou, China). KOD-Plus DNA polymerase was purchased from TOYOBO (Osaka, Japan). The primers and molecular beacons used in the experiments (Supplementary Table S1) were synthesized by Shanghai Sangon Biological Engineering Technology and Services Co., Ltd. (Shanghai, China). One-step RT-qPCR kits were purchased from Medtech. SYBR Gold for resolving urea-denaturing polyacrylamide gel electrophoresis (PAGE) was purchased from ThermoFisher Scientific (Waltham, MA, USA). The full-length RNA of the SARS-CoV-2 (Omicron strain) purified from the virus was obtained from the Hubei Provincial Center for Disease Control and Prevention (HBCDC). Clinical nasopharyngeal and oropharyngeal swab samples from patients infected with SARS-CoV-2 were collected and tested by HBCDC.

### 2.2. Expression and Purification of Recombinant RTX (exo-) and PfAgo 

The recombinant pET-28a (+)-RTX (exo-) was transformed into *E. coli* BL21(DE3) competent cells. One of the positive clones was inoculated into 5 mL LB medium with 50 μg/mL kanamycin and cultured at 220 rpm and 37 °C. Then, the overnight cultures were transferred to 100 mL LB medium with 50 μg/mL kanamycin cultured at 220 rpm and 37 °C until OD_600_ reached to 0.6–0.8. Protein expression was induced by the addition of IPTG at a final concentration of 1 mM. The cultures were incubated at 18 °C for 20 h with continuous shaking at 220 rpm. Then, cells were harvested by centrifugation (15,000× *g*, 5 min, 4 °C) and were resuspended in Buffer I (20 mM Tris/HCl, pH 8.0, 300 mM NaCl, 2 mM MgCl_2_) and lysed by ultrasonic sterilizer (Scientz, Ningbo, China). The cell lysate was centrifuged at 15,000× *g* for 20 min. DNase I and RNase A were added to the supernatant and then was heated at 80 °C for 30 min. The treated supernatant centrifuged at 15 000× *g* for 20 min and was purified with Ni-NTA affinity purification. The column was washed three times using wash buffer (20 mM Tris/HCl, pH 8.0, 300 mM NaCl, 2 mM MgCl_2_, 25 mM imidazole) with 3 times column volume, then target protein was eluted using elution buffer (20 mM Tris/HCl, pH 8.0, 300 mM NaCl, 2 mM MgCl_2_, 250 mM imidazole) with 3 times column volume. The sample was concentrated with a Millipore 50 kD membrane at 4 °C and resuspended in storage buffer (20 mM Tris-HCl, pH 8.0, 300 mM NaCl, 0.5 mM MgCl_2_, 15% [*v*/*v*] glycerol). Bradford Protein Assay Kit was used to determine protein concentrations in this study. Expression and purification of the recombinant PfAgo was performed as previously described [21].

### 2.3. Enzyme Activity Assay of RTX (exo-)

One unit of activity of DNA polymerase is generally defined as the concentration of enzyme required to incorporate 10 nmol dNTPs into the DNA complementary strand in 30 min. In this study, the polymerase activity of RTX (exo-) was measured using Eva EZ™ Fluorometric Polymerase Activity Assay Kit. The commercial KOD-Plus DNA polymerase (TOYOBO) was used as standard control.

### 2.4. Reverse Transcription Assay and PCR Amplification with RTX (exo-)

The reverse transcription (RT) of RTX (exo-) was performed as previously described [18]. The pre-reaction mixture composed 10 pmol of FAM labeled primer (25FAM, Supplementary Table S1), 10 pmol RT-RNA template (Supplementary Table S1), and 400 ng RTX (exo-) protein, reaction at 90 °C for 1 min and then cooled to room temperature. RT with RTX (exo-) was performed as follows: an RTX (exo-) reaction mixture containing 1 μL of the PCR buffer (200 mM Tris-HCl pH 8.8, 600 mM NaCl, 20 mM MgCl_2_, 100 mM (NH4)_2_SO_4_, 200 mM potassium glutamate, 1 mg/mL BSA and 1% Triton X-100), 0.2 μL of dNTP mixture (10 mM dNTPs), 0.2 μL of RiboLock RNase inhibitor (40 U/μL), 1 μL DTT (10 mM) and 5 μL pre-reaction mixture. The mixture was adjusted to 10 μL with RNase-free water. The RT reaction was performed at 68 °C for 30 s in a thermocycler (Bio-Rad, Hercules, CA, USA) as follows: 95 °C for 2 min and then 25 cycles of 95 °C for 10 s (denaturation), 60 °C for 10 s (annealing), 68 °C for 10 s (extension). The positive control with commercial reverse transcriptase enzyme HiFiScript was reacted at 40 °C for 15 min according to the manufacturer’s instructions. The reaction was terminated by adding 25 mM EDTA, then adding 2×TBE-PAGE loading buffer and 100 pmol Blocker (Supplementary Table S1), and performed at 75 °C for 5 min. Finally, 10 μL of the samples were analyzed with 20% TBE-denaturing PAGE.

For the PCR with RTX (exo-) was performed as follows: an RTX (exo-) reaction mixture containing 2.5 μL of the PCR buffer (200 mM Tris-HCl pH 8.8, 600 mM NaCl, 20 mM MgCl_2_, 100 mM (NH4)_2_SO_4_, 200 mM potassium glutamate, 1 mg/mL BSA and 1% Triton X-100), 0.5 μL of dNTP mixture (10 mM dNTPs), 1 μL of RTX (exo-) enzyme (60 ng/μL), 0.5 μL of upstream and downstream primer mixtures (10 μM; Supplementary Table S1), 1 μL λDNA (5 ng/μL). The mixture was adjusted to 25 μL with RNase-free water. The reaction in the classical (basic) setup was performed in a thermocycler (Bio-Rad) as follows: 95 °C for 2 min and then 25 cycles of 95 °C for 10 s (denaturation), 60 °C for 10 s (annealing), 68 °C for 10 s (extension). After the last cycle, a final extension step was added at 68 °C for 10 min. A 5 μL of PCR amplification products were loaded to 1% agarose gel for detection.

### 2.5. The Effect of dNTPs Concentration on the Cleavage Efficiency of PfAgo

The optimal concentration of dNTPs for the PfAgo cleave reaction was determined by adding different concentrations (200 μmol/L, 40 μmol/L, 20 μmol/L, 10 μmol/L and 0 μmol/L) of dNTPs to the PfAgo reaction mixture (2 pmol gDNA (ct-g, Supplementary Table S1), 2 pmol molecular beacon (ct-MB, Supplementary Table S1), 1 μL 10×PfAgo reaction buffer (20 mM HEPES pH 7.5, 250 mM NaCl and 5 mM MgCl_2_), 20 pmol PfAgo protein). After reaction at 95 °C for 5 min, the cleaved products were determined with 20% TBE-PAGE. 

### 2.6. Optimization Conditions of One-Step RTX-PCR with RTX (exo-) 

A single variable was maintained to determine the optimum conditions of one-step RTX-PCR with RTX (exo-), such as the optimum RT time, denaturation and annealing time, denaturation, and annealing temperature. The reaction mixture was prepared as previously described. The optimal RT time for the RTX-PCR reaction was determined by experiments using a time range of 0–5 min at 95 °C; the optimal denaturation and annealing time for the RTX-PCR reaction was determined by experiments using a denaturation and annealing time range of 1–5 s at 95 °C/65 °C; the optimal denaturation temperature was determined using the temperature range of 86–95 °C for optimal denaturation and annealing time; the optimal annealing temperature was determined using the temperature range of 60–72 °C for optimal denaturation and annealing time.

### 2.7. The Recommended Workflow of URPAND

**Figure 1:**
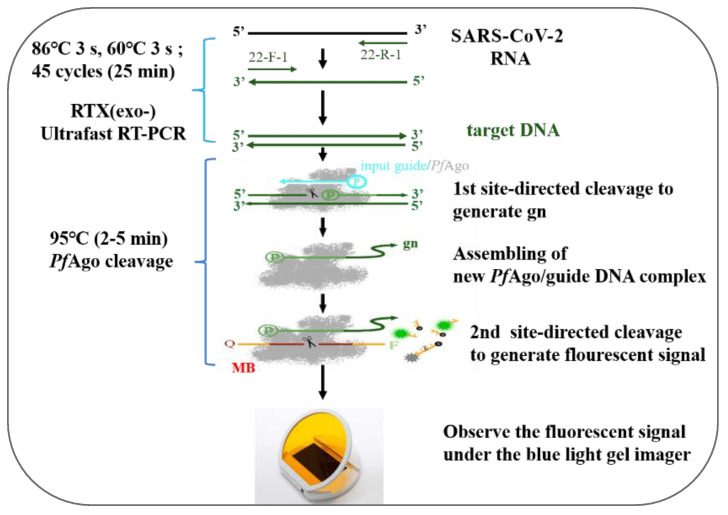
Schematic of recommended URPAND workflow. MB: molecular beacon; gn: newly generated guide.

The URPAND is a detection platform based on Ultra-fast RTX-PCR and PAND, termed URPAND; the workflow of URPAND is shown in Figure 1, briefly, RTX-PCR mixture in a final volume of 25 μL was prepared as previously described. Reaction was performed as follows: 45 thermo-cycles of denaturing at 86 °C for 3 s, extending at 62 °C for 3 s. Subsequently, 1 μL of purified PfAgo (60 pmol), 0.5 μL of 5′-phosphorylated input guide DNA (3 pmol) and 0.5 μL of molecular beacon (10 pmol each) (Supplementary Table S1) and 3 μL of 10×reaction buffer containing 200 mM HEPES (pH 7.5), 2.5 M NaCl, and 3 mM MgCl_2_ was added to the RTX-PCR products to a final volume of 30 μL. The site-directed cleavage was carried out at 95 °C for 2–5 min, followed by detecting the fluorescence intensity of each sample with Blue Light Transilluminators and 20% TBE-PAGE.

### 2.8. The One-Tube URPAND Platform

**Figure 2:**
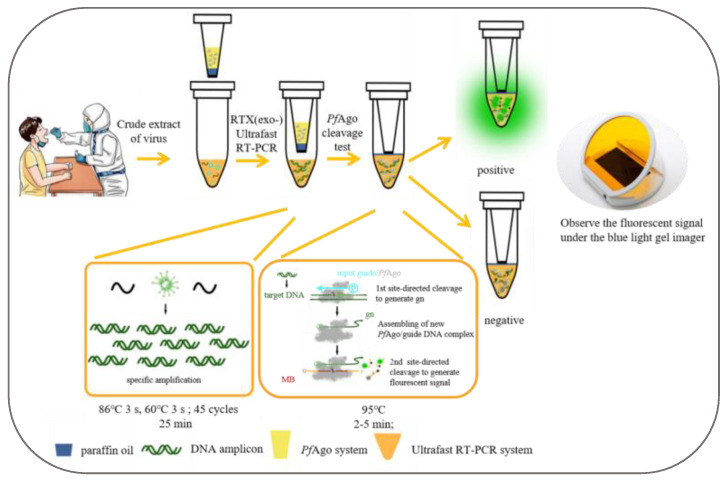
Schematic of One-tube URPAND workflow.

The one-tube URPAND platform is a one-tube detection based on RTX-PCR and PAND; RTX-PCR reaction and PAND reaction mix were simultaneously placed in one-tube. Paraffin wax (58–60 °C) and high-temperature paraffin wax (90–95 °C) were mixed with a mass ratio of 1:1 to form a paraffin mixture; then the paraffin mixture was used as a partition to separate the RTX-PCR mixture and the pfAgo reaction mixture. As shown in Figure 2, the bottom of the truncated pipette tips was sealed with the paraffin mixture, and the pfAgo reaction mixture was handled into the truncated pipette tips; then the pipette tips containing the pfAgo reaction mixture was placed into the PCR tube, which contains the RTX-PCR reaction mixture in the bottom.

The RTX-PCR reaction was performed in a thermocycler as follows: 45 thermo-cycles of denaturing at 86 °C for 3 s, extending at 62 °C for 3 s. The paraffin mixture melted due to the increased temperature during the RTX-PCR reaction, the PNAD mixture was mixed with RTX-PCR products by short centrifugation, then performed at 95 °C for 2–5 min, followed by detecting the fluorescence intensity of each sample with Blue Light Transilluminators.

## 3. Results

### 3.1. Expression and Purification of the RTX (exo-) Protein

**Figure 3:**
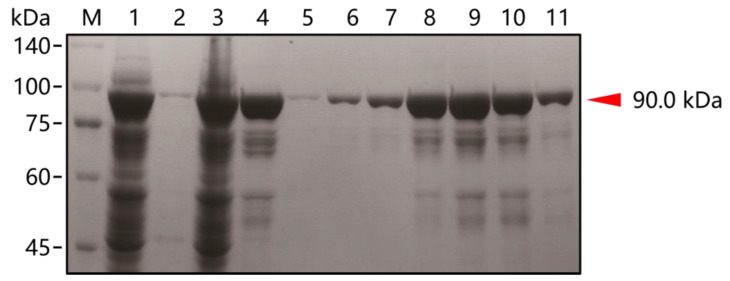
SDS-PAGE analysis of the RTX (exo-) protein expression and purification. M: protein molecular weight standards (the size of each band is indicated on the left). lane 1: whole-cell lysate of RTX (exo-); lane 2: precipitate of cell lysate; lane 3: supernatant of the cell lysate; lane 4: supernatant of cell lysate after heat-treated; lane 5: flow-through sample; lanes 6–11: elution samples with elution buffer of different imidazole concentrations (20, 50, 100, 200, 300 mM). The red arrow indicates the RTX (exo-) protein molecular size (~90.0 kDa).

According to previous studies, RTX (exo-) DNA polymerase has both reverse transcriptase and polymerase activities with the same properties as thermostable. Recombinant strains RTX (exo-) were cultured as described in the methods section. Protein was extracted and analyzed by SDS-PAGE. As shown in Figure 3, the RTX (exo-) (90.0 kDa) was mainly detected in the supernatant of the cell lysate, and miscellaneous proteins were removed when treated with heating. The concentration of RTX (exo-) protein was 3.8 mg/mL, as determined by the Bradford Protein Assay Kit. The Eva EZ™ Fluorometric Polymerase Activity Assay Kit was used to measure the polymerase activity of RTX (exo-). KOD-Plus DNA polymerase (TOYOBO, Osaka, Japan) was used as the standard to draw the enzyme activity standard curve (Figure S1). The estimated activity was 2800 U/mg of purified RTX (exo-) enzyme. Then, the enzyme was dilutedto 0.2 U/μL (60 ng/μL) with the storage buffer and stored at −20 °C for further use.

### 3.2. Reverse Transcription Activity Assay and Amplification Rate Detection of RTX (exo-)

**Figure 4:**
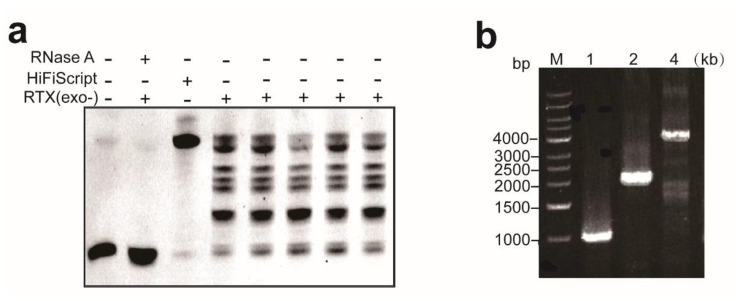
Reverse transcription activity assay and amplification rate detection of RTX (exo-). (**a**) Reverse transcription assay of commercial HiFiScript reverse transcriptase and RTX (exo-); (**b**) PCR with RTX (exo-) to amplify λDNA fragments of different lengths (1 kb, 2 kb, and 4 kb, respectively), the extension time was set as 10 s.

We used a chemically synthesized RNA as a template (Supplementary Table S1) to determine the reverse transcriptase activity of RTX (exo-); we carried out the reverse transcription assay using RTX (exo-) and commercial HiFiScript reverse transcriptase with a FAM-labeled DNA primer. The result is shown in Figure 4a; the negative controls without RTX (exo-) or treated with RNase A could not initialize the reverse transcription, and both the positive control (HiFiScript reverse transcriptase) and RTX (exo-) added individually could complete the process of reverse transcription. The results indicated that the purified RTX (exo-) protein had reverse transcriptase activity. This is consistent with previous reports that RTX (exo-) has reverse transcriptase [18].

In order to verify the amplification rate of RTX (exo-), different primers (Supplementary Table S1) were used to amplify λDNA fragments of different lengths (1 kb, 2 kb, and 4 kb). The PCR extension time was set for 10 s, the PCR products were detected with agarose gel, and the result shown in Figure 4b, RTX (exo-) could amplify 4 kb λDNA fragment in 10 s. The results indicated that the amplification rate of RTX (exo-) was at least 4 kb/10 s, which was a high polymerization rate. The results of reverse transcription and PCR assays demonstrated the purified RTX (exo-) had both reverse transcriptase and polymerase activity, which can be used for subsequent RT-PCR experiments.

### 3.3. The Effect of dNTPs Concentration on the Cleavage Activity of PfAgo 

**Figure 5:**
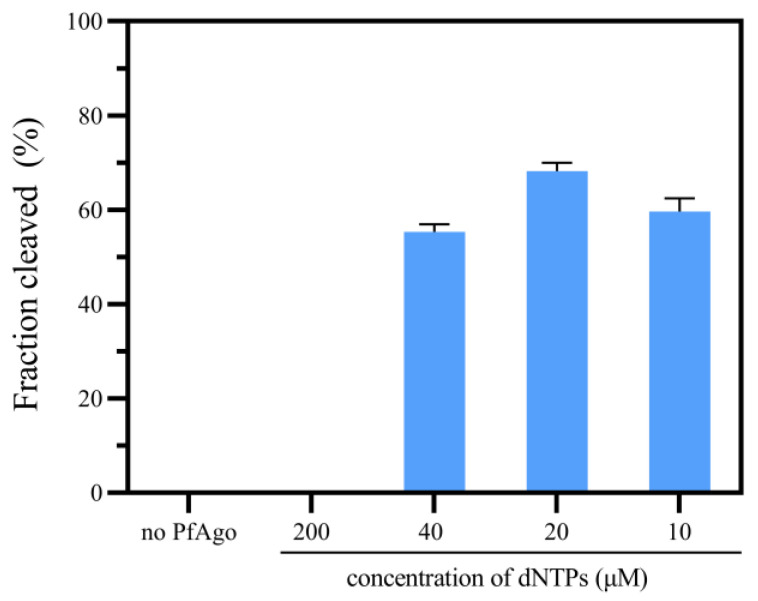
Effect of dNTPs concentration on the cleavage activity of PfAgo assay. no PfAgo: the negative control without PfAgo in reaction mixture. 10–200 μM: different concentration of dNTPs in the PfAgo cleavage reaction. Data represent the mean ± standard error (s.e.); *n* = 3 biological independent experiments.

Research shows that the concentration of dNTPs affects the cleavage efficiency of PfAgo [14]. We used different concentrations of dNTPs in the PfAgo cleavage reaction to explore the optimal dNTPs concentration with 200 μM, 40 μM, 20 μM, and 10 μM. As shown in Figure 5, PfAgo demonstrated high activity when the concentration of dNTPs was 20 μM, which was optimal. However, when the concentration of dNTPs reached 40 μM or even more, the cleavage efficiency of PfAgo decreased, even causing a loss of activity when the concentration of dNTPs reached 200 μM. We hypothesize that the high concentration of dNTPs may inhibit the catalytic active site, resulting in pfAgo being unable to bind the guide or target. Thus, pfAgo’s activity was blocked. While the dNTPs in the RTX-PCR system were consumed by target amplification, the inhibition was discharged. This indicates that, to ensure the sensitivity and the accuracy of URPAND, the concentration of dNTPs should be optimized depending on the target amplification efficiency.

### 3.4. The Optimal Conditions of RTX-PCR Based on RTX (exo-)

In order to simplify steps and reduce the time of RTX-PCR with RTX (exo-), we optimized RTX-PCR from reverse transcription time, denaturation and annealing times, and temperatures. A single variable was used to optimize the RTX-PCR amplification reaction. We used SARS-CoV-2 RNA samples (provided by the Hubei Provincial Center for Disease Control and Prevention) as RNA templates of RTX-PCR based on RTX (exo-) to amplify an 88 bp DNA fragment product. 

**Figure 6:**
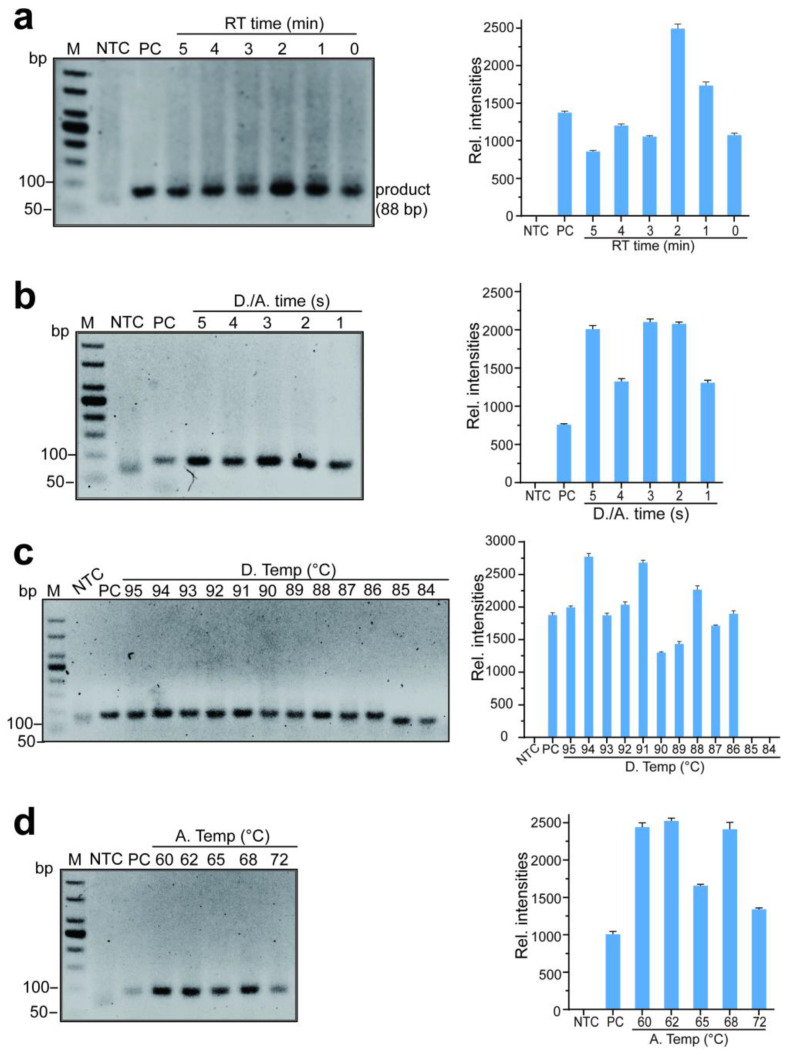
Optimization of RTX-PCR conditions with RTX (exo-). Estimation of the shortest reverse-transcription step (**a**), RTX-PCR with different denaturation and annealing time (**b**), the optimal denaturation temperature (**c**), and annealing temperature (**d**) in RTX-PCR. The assays were performed with RTX (exo-) using purified SARS-CoV-2 RNA as a template to amplify an 88 bp specific amplicon of the N gene of SARS-CoV-2. NTC: negative control without RNA template; PC: positive control with commercial one-step RT-PCR kit (Yeasen); RT time: reverse transcription time; **D./A. time**: denaturation and annealing time; D. Temp: denaturation temperature; A. Temp: annealing temperature. For (**a**–**d**): left, representative gel electrophoresis analysis of the RTX-PCR products; right, the relative intensities of RTX-PCR products were quantified using ImageJ, data represent the mean ± standard error (s.e.); *n* = 3 biologically independent experiments.

Therefore, to investigate if the time of the reverse transcription (RT) step could be shortened, the RTX-PCR assay was performed with an RT step time of 5 min, 4 min, 3 min, 2 min, 1 min, and 0 min, respectively, then followed by the PCR program (denatured at 95 °C, 5 s; annealing at 65 °C, 5 s) steps of 45 cycles, and the amplification products were detected with 3% agarose gel. As shown in Figure 6a, even if the RT step was 0 min, adequate RTX-PCR products could be detected, indicating that the RT process had been completed during the process of denaturation at 95 °C. The RT step can be removed in RTX-PCR with RTX (exo-) when amplifying short fragments. 

To further shorten the RTX-PCR time, we optimized the PCR program. Firstly, the denaturing and annealing temperatures were set as 95 °C and 65 °C, respectively, and the denaturation and annealing time (D./A. time) were set as 5 s, 4 s, 3 s, 2 s, and 1 s. As shown in Figure 6b, the experiment with D./A. time of 3 s was sufficient to amplify adequate RTX-PCR products, and the number of products did not significantly increase, though, with time extension. Thus, the amplification conditions of 95 °C for 3 s and 65 °C for 3 s were chosen for subsequent experiments. Heating and cooling processes will increase PCR time during the annealing and denaturation steps in PCR. Therefore, we explored the influence of the annealing and denaturation temperatures on RTX-PCR with RTX (exo-). We set annealing at 65 °C (3 s), and the denaturation process was carried out at temperatures of 95 °C, 94 °C, 93 °C, 92 °C, 91 °C, 90 °C, 89 °C, 88 °C, 87 °C, 86 °C, 85 °C and 84 °C for 45 cycles. As shown in Figure 6c, the denaturation temperature at 86 °C was sufficient to amplify enough RTX-PCR products, and when the denaturation temperature was dropped to 85 °C or lower, there was no obvious target product. In the process of PCR, it takes time to heat up and cool down; when the denaturation temperature was selected at a relatively low temperature, it can reduce the time of heating up and cooling down, thereby the time of the PCR process is reduced; thus, 86 °C was chosen for the denaturation process. Then, the annealing processes were optimized with 60 °C, 62 °C, 65 °C, 68 °C, and 72 °C. As shown in Figure 6d, 62 °C was the optimal temperature for annealing.

Therefore, the optimal RTX-PCR condition for URPAND was: denatured at 86 °C for 3 s; annealing at 62 °C for 3 s, steps of 45 cycles, then the time of the RTX-PCR amplification step was shortened from 60 min to 25 min.

### 3.5. The Feasibility and Validation of RTX-PCR in URPAND

**Figure 7:**
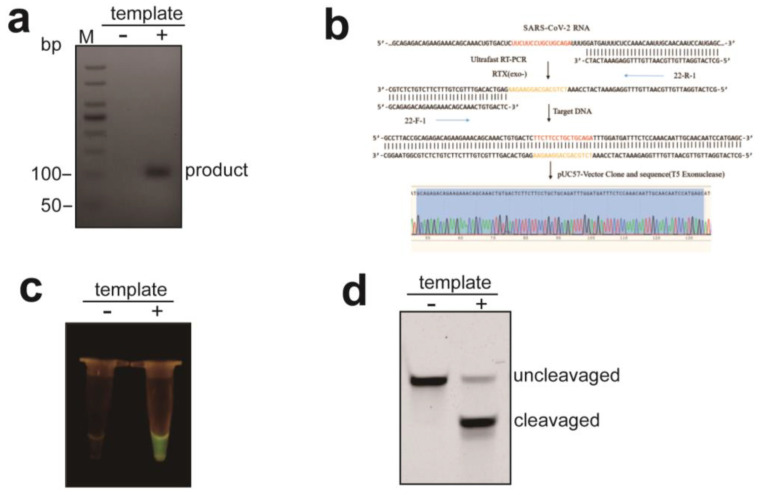
The feasibility and validity verification of URPAND. (**a**): The RTX-PCR products were detected with 3% agarose gel electrophoresis. (**b**): Sequencing analysis of the RTX-PCR products. Testing results of RNA sample with URPAND by fluorescence signal detecting with Blue Light Transilluminators (**c**) and with TBE-denaturing PAGE (**d**).

To evaluate the feasibility and validation of URPAND, we amplify an 88 bp DNA fragment of the N gene of SARS-CoV-2. The coding region of the N gene in the viral genome (GenBank Accession No. MN985325.1) was chosen as the target in this study. The key point of URPAND is the efficient cleavage of PfAgo/input guide complex to the target DNA. Therefore, we designed 12 guides covering the full amplifying region of the N gene and tested the cleavage activity of corresponding PfAgo/guide complexes to the ssDNA targets; accordingly, guide 7-g5 was chosen for URPAND due to the high activity of the complexes (Figure S2). As shown in Figure 7a, there was an amplification product only in the presence of a template. The PCR products were further sequenced, and the sequence of PCR products was constant to the target sequence, as shown in Figure 7b. The PCR product could be cleaved by PfAgo with the optimized input gDNA (7-g5) to serve as a new guide DNA (generating gDNA) binding to PfAgo to cleave the complementary molecular beacon to produce a fluorescence signal [14] under a Blue Light Transilluminators as shown in Figure 7c. Further, to confirm the molecular beacons were whether cleaved by PfAgo, the products were detected with TBE-denaturing PAGE, as shown in Figure 7d; the molecular beacons could only be cleaved with templates added to the RTX-PCR reaction mixture, which is consistent with the result of fluorescence test shown as Figure 7c. 

### 3.6. The Sensitivity and Specificity of URPAND

**Figure 8:**
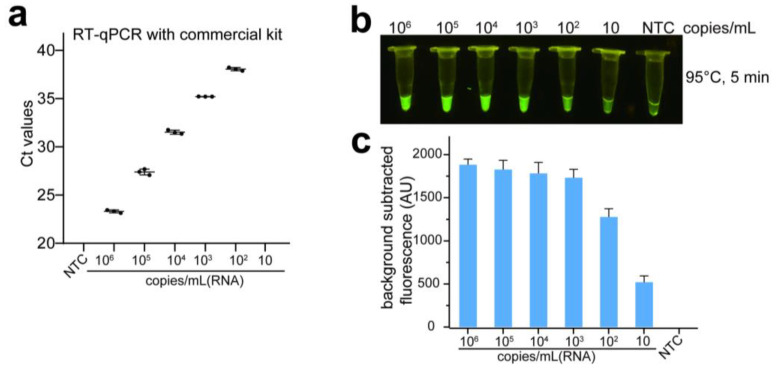
LoD for RT-qPCR detection kit from Medtech (**a**) and URPAND assay (**b**), the fluorescence intensity of URPAND assay (**c**). NTC: negative control without RNA template. For (**a**,**b**), data represent the mean ± standard error (s.e.); *n* = 3 biological independent experiments.

The analytic limit of detection (LoD) of URPAND was compared with the SARS-CoV-2 nucleic acid RT-qPCR detection kit from Medtech with the N gene as the target. The SARS-CoV-2 RNA samples were diluted with non-ribozyme water from 10^6^ copies/mL to 10 copies/mL. As shown in Figure 8a, when the target was further diluted to 100 copies/mL, Ct-values with three replicates of each titer were >38, which was the gray area of qPCR, and RT-qPCR failed to detect the target of 10 copies/mL (Figure 8a), this was consistent to that the LoD of kits developed by different companies ranges from 100 copies/mL to 1000 copies/mL [24], the Ct-values increased as the copy numbers decreased and were not detected when the template concentration was decreased to 10 copies/mL. In comparison with the RT-qPCR assay, the sensitivity of URPAND was much higher. When the target was further diluted to 10 copies/mL, URPAND could still generate significant fluorescence (Figure 8b,c). The results demonstrated that the sensitivity of URPAND is equivalent to or even higher than the commercial RT-qPCR detection kit.

**Figure 9:**
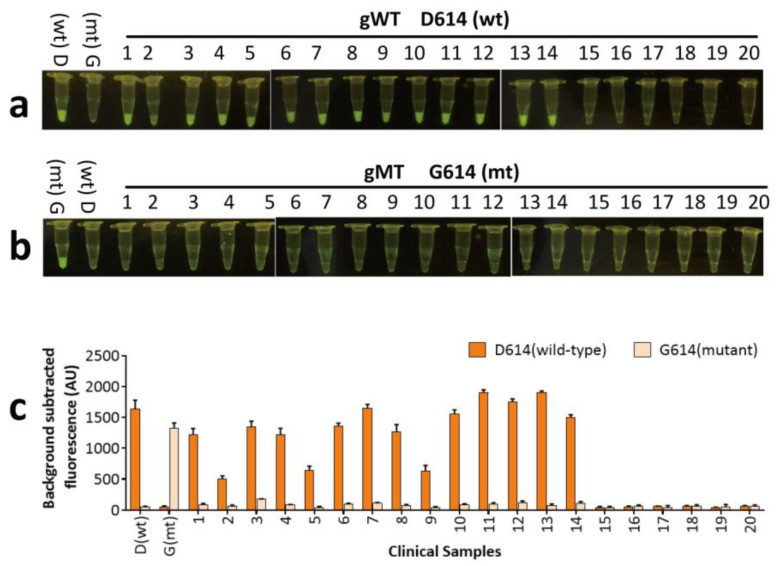
Identifying SARS-CoV-2 D614G mutants with URPAND in clinical samples. (**a**) testing results of clinical samples collected from February to May 2020 with gMT; (**b**) testing results of clinical samples collected from February to May 2020 with gWT; (**c**) detection of nt23403 SNP in clinical samples with URPAND.

pfAgo-based detection was able to identify a single nucleotide mutation that could be used to distinguish the D614G mutant of SARS-CoV-2 [14]. To determine the specificity of URPAND, the D614G mutant was detected with URPAND according to the SARS-CoV-2-PAND [14], except that the RT-PCR was instead of RTX-PCR. As shown in Figure 9, URPAD was able to distinguish mutant and wild-type distinctly by comparing the difference between the readouts. Fourteen clinical positive samples (1–14) collected from February to May 2020 were all defined as wild type (D614). This result was consistent with previous reports [14,25], and six clinical negative samples stimulated no obvious fluorescence with neither gWT nor gMT.

### 3.7. The Sensitivity Assay and Feasibility Analysis of “One-Tube” URPAND

**Figure 10:**
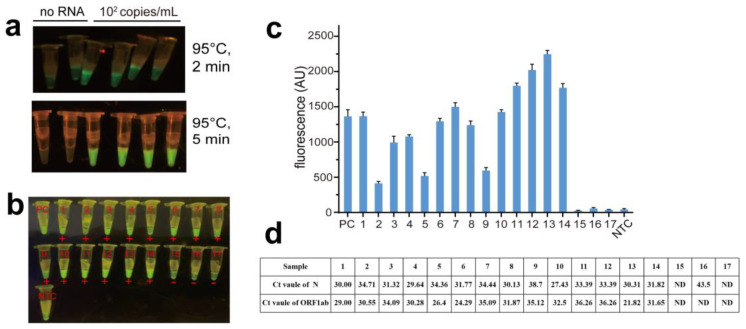
The “one-tube” URPAND assay with SARS-CoV-2 RNA samples and clinical samples. (**a**): one-tube-URPAND detection of SARS-CoV-2 RNA for 2 and 5 min. (**b**): Identifying SARS-CoV-2 clinical samples with “one-tube” URPAND by naked eyes. PC: positive control with commercial one-step RT-PCR kit (Yeasen); NTC: negative control without RNA template; 1–14: Clinical positive samples; 15–17: Clinical negative samples. (**c**): the fluorescence intensity of the clinical samples. (**d**): the Ct Value of the clinical samples. data represent the mean ± standard error (s.e.); *n* =  3 biological independent experiments.

The “one-tube” detection RNA samples ability of the assay to perform RTX-PCR and PfAgo cleavage in one tube would be highly desirable, especially for routine testing of a large number of samples, one-tube SARS-CoV-2 detection platform can avoid aerosol contamination. Therefore, we performed a “one-tube” URPAND experiment as the method described. We used 100 copies/mL diluted SARS-CoV-2 RNA samples for the detection; there was a significant fluorescence in 2 min and more conspicuous in 5 min compared to negative control in “one-tube” URPAND (Figure 10a). This indicated that the “one-tube” URPAND is feasible to detect the low concentration of SARS-CoV-2 RNA. The results emphasized that “one-tube” URPAND demonstrated higher sensitivity, detecting as low as 100 copies/mL, which is equivalent to or higher than the commercial RT-qPCR kit.

### 3.8. Analysis of Clinical Samples with “One-Tube” URPAND

Further, “one-tube” URPAND was also applied to identify fourteen clinical positive and three clinical negative samples confirmed by RT-qPCR (Figure 10d); all the results were negative for COVID-19 infection (Figure 10b,c), which were consistent with the results of RT-qPCR test carried out (Ct > 40).

## 4. Discussion

Expensive equipment and long detection time are limitations of the RT-qPCR platform. The currently available PfAgo-based detection platform requires separation of the amplification and detection steps, increasing the complexity of the operation and the risk of aerosol contamination [14,15,22]. Here, we developed a “one-tube” URPAND method by coupling RTX-PCR with pfAgo. which is a reliable alternative to the gold standard RT-qPCR technique. URPAND achieved an ultra-fast RTX-PCR process based on a thermostable RTX (exo-) with both reverse transcriptase and DNA polymerase activity; with the optimal PCR program, the time of RTX-PCR was reduced to 25 min, and the time of pfAgo cleavage reaction was 2–5 min. Therefore, “one-tube” URPAND was able to detect nucleic acids within 30 min. The sensitivity of URPAND, in comparison with RT-qPCR using commercial kits, is that the URPAND has a LoD of 10 copies/mL, with a much higher sensitivity and less time cost [26]. The limit of detection is particularly critical as the purpose of testing is to diagnose the virus at early infection rather than the time of symptom onset, which for COVID-19 starts from 104 copies/mL [27]. Most newly developed methods have a limit of detection at 100 copies/mL or less, which corresponds to the viral detection at 2–3 days before the onset of symptoms [27,28]. Whether URPAND could detect the virus in the early (pre-)symptomatic phase should be further measured.

The “one-tube” URPAND presented here is one of the powerful tools to enable rapid diagnosis without trained personnel and expensive equipment to detect SARS-CoV-2; only two enzymes were used in the system, both RTX (exo-) and pfAgo were thermotolerant, which could be purified simply and cost-effectively. Although the current epidemic situation is controlled, the diagnosis with minimal equipment, cost-effective regents, and fast turnaround time can be widely used in local emergency departments, clinics, airports, stations, homes, and other field locations, which can be key to responding to emergencies. This is particularly crucial for low- and middle-income countries to respond to health crises with greater autonomy and at lower economic costs.

While our study was limited to 20 clinical samples, future work should involve a larger sample size for enhanced validation. Additionally, the method can operate in a simple thermo cycler device with rapid ramp rates; thus, detection time could be further reduced. Combined with a mobile application that could distinguish the fluorescence or lateral flow strips, the “one-tube” URPAND could be designed for point-of-care testing (POCT).

## Data Availability

All relevant data of this study are presented. Additional data will be provided upon request.

## Supplementary Materials

The following supporting information can be downloaded at: https://www.mdpi.com/article/10.3390/bios14050245/s1; Figure S1: The standard to draw the enzyme activity standard curve; Figure S2: Screen analysis of input gDNA for efficient cleavage of the N gene by PfAgo; Table S1: primers, guides, and molecular beacons used in this study.
