# Supplementary material for: A Fast and Sensitive One-Tube SARS-CoV-2 Detection Platform Based on RTX-PCR and Pyrococcus furiosus Argonaute
Source: Biosensors (Basel). 2024 May 13;14(5):245. doi: 10.3390/bios14050245 (PMC11118887; doi:10.3390/bios14050245)
Supplement: Supplementary file 1 [file biosensors-14-00245-s001.zip › biosensors-2941878-supplementary.pdf]

**Supplementary**

Supplementary Table 1 primers， guides and molecular beacons used in this study

| **Name of the Primers, guides and molecular beacons** | **Sequence (5′-3′)** |
|---|---|
| Primers used for testing the reverse transcriptase and DNA polymerase of RTX (exo-) | |
| 25FAM | 6-FAM-CCCTCGCAGCCGTCCAACCAACTCA |
| RT-RNA | CGAAAACGGCAUUAUAUGAUAUCUUGUGAGUUGGUUGG ACGGCUGCGAGGG |
| Blocker | CCCTCGCAGCCGTCCAACCAACTCACAAGATATCATATAAT GCCGTTTTCG |
| λDNA-F | GGCCTCTGTCGTTTCCTTTCTCTG |
| λDNA-1R | GGTGAGCGTGTTATCCCGGTG |
| λDNA-2R | CCGTAGACGGATGCCCCTTTAATG |
| λDNA-4R | GCATCACCGCTTCCTGAACTTC |
| Primers, guides and molecular beacons used for targets of N gene | |
| 4-f | AATAAGCATATTGACGCATACAAAACATTCCCACC |
| 4-r | TCTGTCTCTGCGGTAAGGCTTGAGTTTCATC |
| 4-g1 | CTTTTTAGGCTCTGTT |
| 4-g2 | GTCCTTTTTAGGCTCT |
| 4-g3 | TTTGTCCTTTTTAGGC |
| 4-g4 | CTTTTTGTCCTTTTTA |
| 4-g5 | CTTCTTTTTGTCCTTT |
| 4-g6 | CTTCTTCTTTTTGTCC |
| 4-g7 | AGCCTTCTTCTTTTTG |
| 7-f | TCAAGCCTTACCGCAGAGACAGAAGAAACAGC |
| 7-r | GCTCATGGATTGTTGCAATTGTTTGGAGAAATCATCC |
| 7-g1 | AGAAGAGTCACAGTTT |

| | |
|---|---|
| 7-g2 | GGAAGAAGAGTCACAG |
| 7-g3 | GCAGGAAGAAGAGTCA |
| 7-g4 | GCAGCAGGAAGAAGAG |
| 7-g5 | TCTGCAGCAGGAAGAA |
| ct-g | GTTTGGTGGATGTGGA |
| 22-F-1 | GCAGAGACAGAAGAAACAGCAAACTGTGACTC |
| 22-R-1 | GCTCATGGATTGTTGCAATTGTTTGGAGAAATCATC |
| ct-MB | 6-FAM-tttacgCATCCACATCCACCAAACGTAATGcgtaaa-BHQ1 |
| 7-5MB | 6-FAM-cgcaccTCCAAATCTGCAGCAGggtgcg-BHQ1 |
| 614-F | CAAATACTTCTAACCAGGTTGCTGTTC |
| 614-R | GCAACAGGGACTTCTGTGC |
| gWT | GATGTTAACTGCACAG |
| gMT | GGTGTTAACTGCACAG |
| MB-614 | 5' FAM-cgcaccTATCAGGATGTTAACTggtgcg-BHQ1-3' |

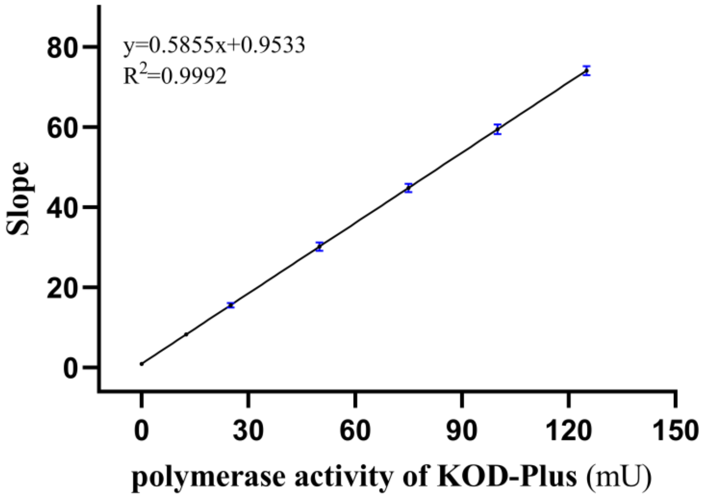


Figure S1. Titration of KOD-plus DNA Polymerase Activity determined with Eva EZ™ Fluorometric Polymerase Activity Assay Kit.

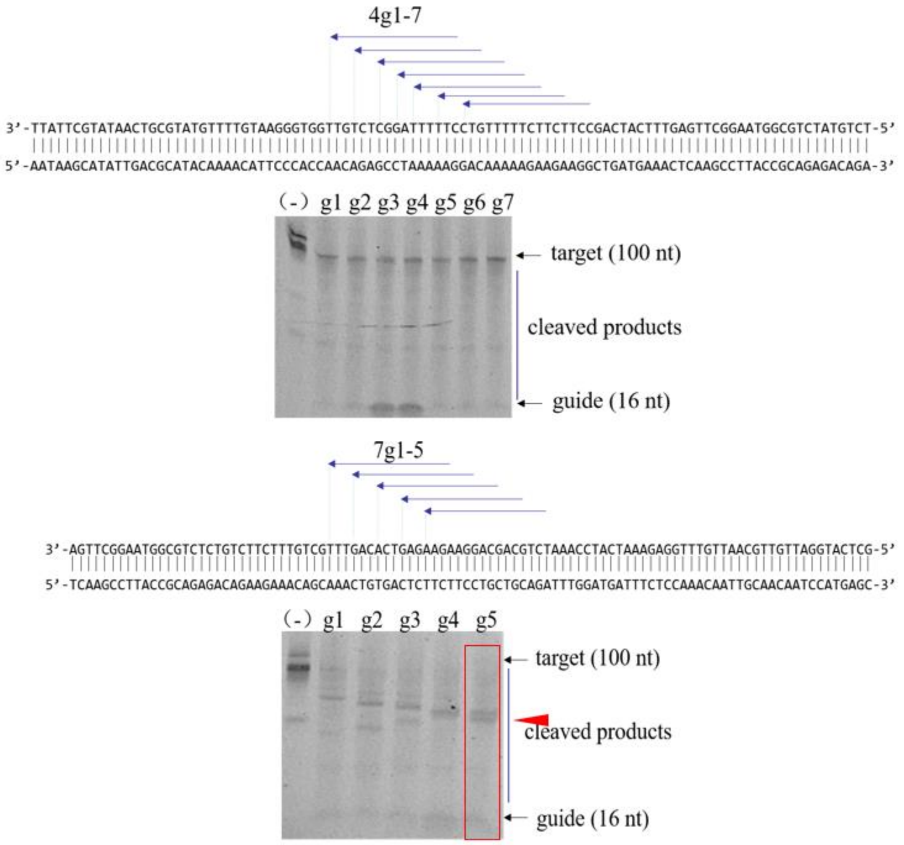


**Figure S2.** Screen analysis of input gDNA for efficient cleavage of the N gene by PfAgo.Guides 4g1-7 cover the first fragment while 7g1-5 cover the second fragment. The boundary of each guide is indicated on the top of the sequences. Urea-denaturing PAGE was performed to analyze the cleavage of the targets.
